# A new method for visualizing cerebrovascular vessels

**DOI:** 10.3389/fnmol.2026.1753542

**Published:** 2026-06-01

**Authors:** Qiang Gu, James Raymick, Sumit Sarkar, Jyotshna Kanungo

**Affiliations:** Division of Neurotoxicology, National Center for Toxicological Research, U.S. Food and Drug Administration, Jefferson, AR, United States

**Keywords:** astrocyte, autofluorescence, blood vessels, blood–brain barrier, cerebrovasculature, microglia, neurovascular unit, lectin histochemistry

## Abstract

Studying the cerebrovasculature is crucial for both basic neuroscience and clinical medicine, as the brain’s vascular network is dynamic and undergoes changes with aging and disease. In this study, we introduce a novel approach for visualizing cerebrovascular vessels. The method is simple, requires no exogenous fluorophores, and allows for multiple labeling with additional molecular markers. This method will facilitate cerebrovascular research concerning angiogenesis and remodeling associated with aging, cerebrovascular diseases, and neurodegenerative disorders.

## Introduction

The cerebrovasculature serves to maintain and regulate brain homeostasis and function by supplying oxygen, nutrients, and other critical elements and by removing waste products (Cipolla, 2009). As a physical infrastructure, the brain’s vascular network is not static, and the vascular architecture can change based on need and the local environment, particularly during brain development, aging, and pathological conditions such as cerebrovascular diseases, neurodegenerative disorders, brain neoplasms, traumatic brain injuries, and epilepsy (Bogorad et al., 2019; Zedde and Pascarella, 2024; Marchi and Lerner-Natoli, 2013). Therefore, examining cerebrovascular changes associated with disease onset and progression can help improve our understanding of the causes, consequences, and underlying cellular and molecular mechanisms of these changes and can aid in developing clinical intervention options (Deshpande et al., 2025).

Blood autofluorescence is a well-known phenomenon that has been applied in biomedical and clinical research (for a comprehensive review, see Shrirao et al., 2021). It has been shown that blood autofluorescence can be used to identify blood vessels in murine and human airways using multiphoton microscopy (Kretschmer et al., 2016). In addition, Li et al. (2020) demonstrated the use of blood autofluorescence to identify arteries and veins in the mouse cerebral cortex using two-photon microscopy. In this study, a new approach for visualizing cerebral vessels is presented that is based on blood autofluorescence and is completely different from any previously published methods. Since blood is retained *in situ*, this method allows visualization of the cerebrovasculature without using external artificial fluorophores. Compared with conventional vessel staining methods that use immunofluorescent or histochemical labeling or involve filling vessels with specific chemicals, the present method is simpler, does not require external fluorophores or tracers, and saves on materials, cost, labor, and time.

## Methods

Fischer-344 rats were purchased from Envigo (Indianapolis, IN, USA) and bred in-house. Postmortem human brain tissue was obtained from the National Institutes of Health (NIH)-funded NeuroBioBank at the University of Maryland Brain and Tissue Bank. The use of the animals and the postmortem human brain samples was approved by the Institutional Animal Care and Use Committee and the Institutional Review Board, respectively.

Adult rats were euthanized with Euthasol (pentobarbital, 100 mg/kg, i.p.) (Virbac AH, Fort Worth, TX, USA), followed by decapitation. The brain was quickly removed, submerged in 4% paraformaldehyde (PFA) (Sigma-Aldrich, St. Louis, MO, USA), and stored in PFA at 4 °C. For brain samples without blood retention, the rats, following pentobarbital euthanasia, underwent transcardial perfusion with phosphate-buffered saline (PBS) (Gibco, Grand Island, NY, USA), followed by 4% PFA and the same subsequent procedures as those of non-perfused samples. After cryoprotection in 30% sucrose (Fisher Scientific, Fair Lawn, NJ, USA), their brain tissues were cut into sections (25–50-μm thickness) using a cryostat (Leica CM3050S, Nussloch, Germany) and washed in PBS. The tissue sections were incubated in a medium containing 15% (v/v) N-butyldiethanolamine (MilliporeSigma, Burlington, MA, USA) and 10% (v/v) (Triton X-100 Sigma-Aldrich, St. Louis, MO, USA), which are components of a tissue clearing protocol (Matsumoto et al., 2019), at 37 °C for 6–8 h. Because this is a chemical clearing process, thicker sections require more time than thinner sections. The tissue sections were washed in 0.1 × PBS and mounted onto slides, dehydrated, and coverslipped.

For multiple fluorescence labeling, following the incubation procedure described above, the brain sections were washed in PBS for 3 × 5 min, incubated in 5% normal goat serum (NGS) (MP Biomedicals, Solon, OH, USA) at room temperature for 20 min, and then incubated in a primary antibody medium containing PBS, 1% NGS, 0.1% Kodak PhotoFlo-200 (Rochester, NY, USA), chicken anti-rat glial fibrillary acidic protein (GFAP) antibody (MilliporeSigma, Burlington, MA, USA) (1:1000), and rabbit anti-rat Ionized calcium-binding adapter molecule 1 (IBA1) antibody (Invitrogen, Carlsbad, CA, USA) (1:500) at 4 °C overnight. The tissue sections were washed in PBS for 3 × 5 min, incubated in secondary antibody medium containing PBS, 1% NGS, 0.1% Kodak PhotoFlo-200, and biotinylated goat anti-chicken IgG (Jackson ImmunoResearch Laboratories, West Grove, PA, USA) (1:200) for 2 h. The tissue sections were washed again in PBS for 3 × 5 min, and incubated in a third antibody medium containing PBS, 1% NGS, 0.1% Kodak PhotoFlo-200, Cy5-conjugated anti-rabbit IgG (Jackson ImmunoResearch Laboratories, West Grove, PA, USA) (1:100), and rhodamine-conjugated streptavidin (Jackson ImmunoResearch Laboratories, West Grove, PA, USA) (1:200). After washing with PBS for 5 min, the tissue sections were incubated in PBS containing 1:10,000 4’,6-diamidino-2-phenylindole (DAPI) (Invitrogen, Carlsbad, CA, USA) for 5 min, washed in PBS for 3 × 5 min, and then briefly rinsed in 0.1x PBS, following which they were mounted onto slides, dehydrated, and coverslipped.

For lectin histochemistry, the tissue sections were washed in PBS and then incubated in 100 mM glycine (Fisher Scientific, Fair Lawn, NJ, USA) for 15 min. After additional washing with PBS, the sections were incubated in PBS containing 1% bovine serum albumin (BSA) (Sigma-Aldrich, St. Louis, MO, USA) for 60 min, transferred to a PBS solution containing 1% BSA, 0.05% Triton X-100, and 5 μg/mL fluorescein isothiocyanate (FITC)- or DyLight594-conjugated tomato lectin, (Invitrogen, Carlsbad, CA, USA; Vector Laboratories, Newark, CA, USA, respectively) followed by overnight incubation at 4 °C. After washing with PBS, the tissue sections were mounted onto slides, dehydrated, and coverslipped.

Optical images were captured using a Nikon Eclipse Ni microscope (Tokyo, Japan) or a Zeiss Axioscan-7 slide scanner (Jena, Germany).

## Results

**Figure 1:**
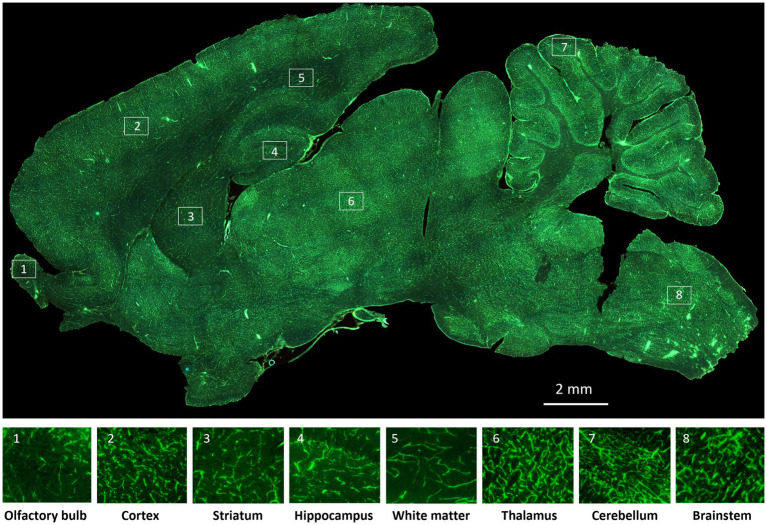
A parasagittal section of a rat brain showing numbered, equal-sized rectangles in different regions and the corresponding zoomed-in images of vessels.

**Figure 2:**
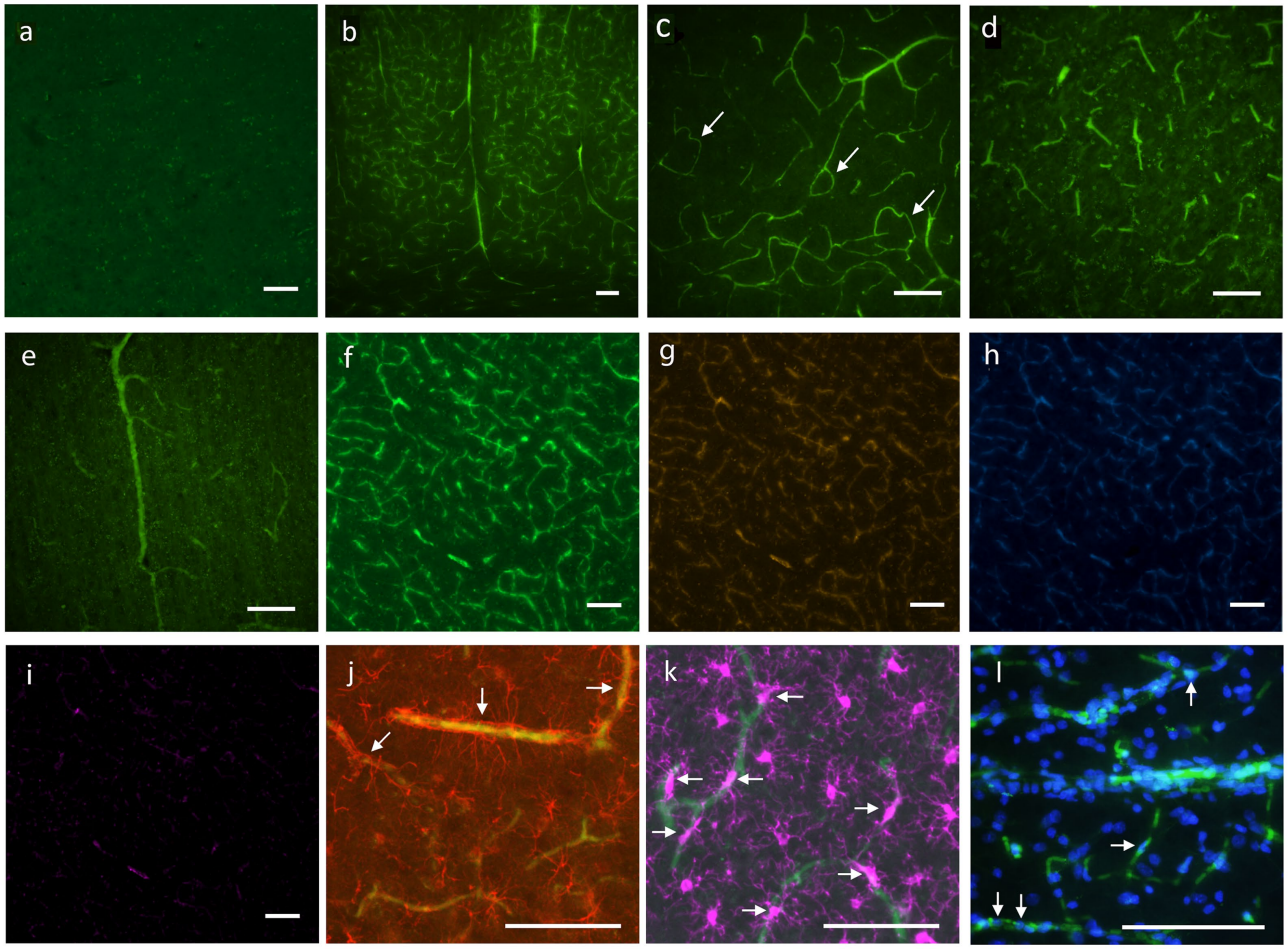
**(a)** A rat cortex without blood preservation. **(b)** Fine capillaries and thick vessels with branches and **(c)** loop-shaped vessels (arrows) in the rat cortex. **(d)** Capillaries in the frontal cortex and **(e)** a long vessel in the white matter of an 82-year-old woman. Vessels in the rat cortex were imaged with different filter sets: FITC **(f)**, Cy3 **(g)**, DAPI **(h)**, and Cy5 **(i)**. **(j)** Co-labeling of vessels (green) with GFAP (red) in the rat cortex showing processes intertwined with vessels (arrows). **(k)** Co-labeling with IBA1 (purple) in the rat cortex showing cell bodies (arrows) attached to vessels (green) and processes intertwined with vessels. **(l)** Co-labeling of vessels (green) with DAPI (blue) in the rat cortex showing nuclei co-localized with vessels (arrows) and surrounding vessels. Scale bars = 100 μm.

Figure 1 shows vessel images from a rat brain, with apparent interregional differences. Examples of fine capillaries and thick vessels with branches, along with unique loop-shaped structures, are displayed in Figure 2. The images demonstrate the effectiveness of this method without using any external fluorophore. A fundamental requirement of this method is to retain blood *in situ*. When blood is removed from the brain, the vessel structure is no longer visible (Figure 2a), suggesting that vessel signals are derived from the endogenous autofluorescence associated with the blood. This requirement makes this method particularly suitable for vessel analysis in postmortem human brains, which, for ethical reasons, are commonly preserved with blood remaining *in situ*. To validate this possibility, postmortem brain tissues from six human subjects of various ages and postmortem processing intervals were obtained from an NIH-sponsored brain bank and processed following the same methodological procedure. The results show that vessels could indeed be visualized in human brains without applying any external fluorophore (Figures 2d,e), thereby confirming the applicability of this method for visualizing vessels in postmortem human brains.

Consistent with previous observations of blood autofluorescence (Shrirao et al., 2021), endogenous vessel signals appear to span a broad spectrum and are visible through conventionally used optical filters but with different emission intensities in the following descending order: 515–565 nm (FITC), 570–640 nm (Cy3), 412–452 nm (DAPI), and 673–712 nm (Cy5) (Figures 2f–i). Therefore, the FITC filter is most suitable for visualizing endogenous autofluorescence associated with the vessels.

To demonstrate that this method is compatible and useful for multiple fluorescence labeling and co-localization studies, immunocytochemistry using GFAP and IBA1 antibodies and histochemistry using DAPI were conducted. GFAP immunolabeling shows vessels wrapped by astrocyte processes (Figure 2j), whereas IBA1 shows vessels wrapped by microglial processes and microglia attached to vessels (Figure 2k). DAPI labeling shows colocalization of nuclei and vessels (Figure 2l). These results demonstrate that this method can be combined with other cellular and molecular markers to facilitate mechanistic investigations of vessel development, remodeling, and plasticity.

**Figure 3:**
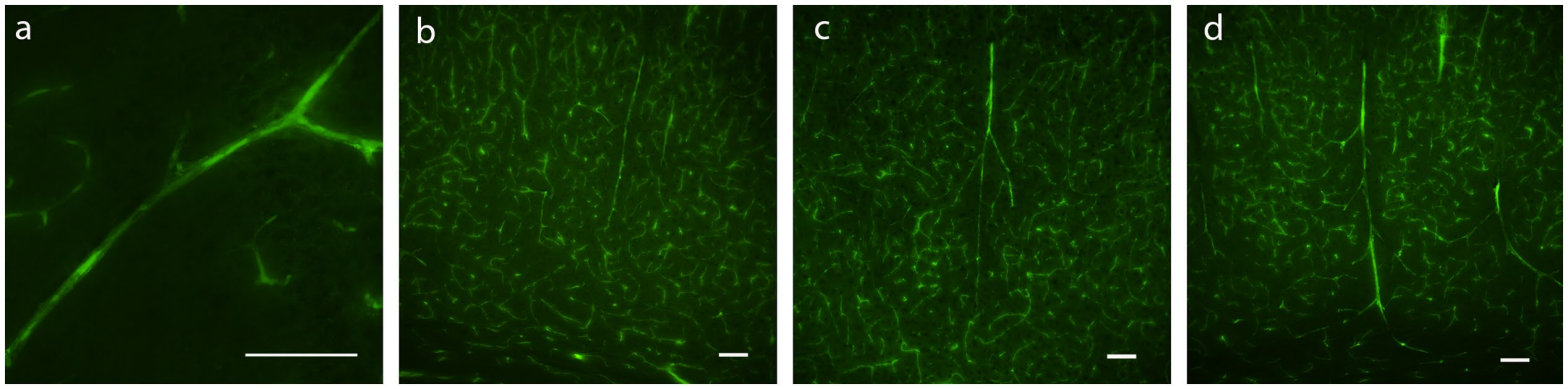
**(a)** A high-magnification image showing vessels with an uneven distribution of autofluorescence intensities. **(b)** A rat cortex that was processed after being maintained in fixative for 13 months. **(c)** A rat cortex that was processed after first being sectioned and then maintained in PBS at 4 °C for 10 months. **(d)** A rat cortex section was processed, and the slide was kept in the dark at room temperature for 10 months. For comparison, an image of the same area acquired immediately after tissue processing 10 months earlier is presented in Figure 2b. Scale bars = 100 μm.

It can be observed that, while blood continuously fills the vessels, the autofluorescence intensities are not homogeneous within the vessels (Figure 3a). However, as can be seen in Figure 3a, the uneven autofluorescence intensities in some locations do not affect the identification of vessels and their morphological structures.

To evaluate the stability of the autofluorescence signals, the following experiments were conducted: (1) processed brain samples stored in fixative for up to 13 months (the longest available storage time at the time of testing), (2) processed brain sections maintained in PBS at 4 °C for up to 10 months (the longest available storage time at the time of testing), and (3) re-examined previously processed and coverslipped brain section slides after storage in the dark at room temperature for up to 10 months (the longest available storage duration at the time of testing). The results reveal that, despite these different storage conditions and time intervals, autofluorescence signals remain visible and robust (Figures 3b–d), suggesting that autofluorescence signals are relatively stable and resistant to fading after fixation.

**Figure 4:**
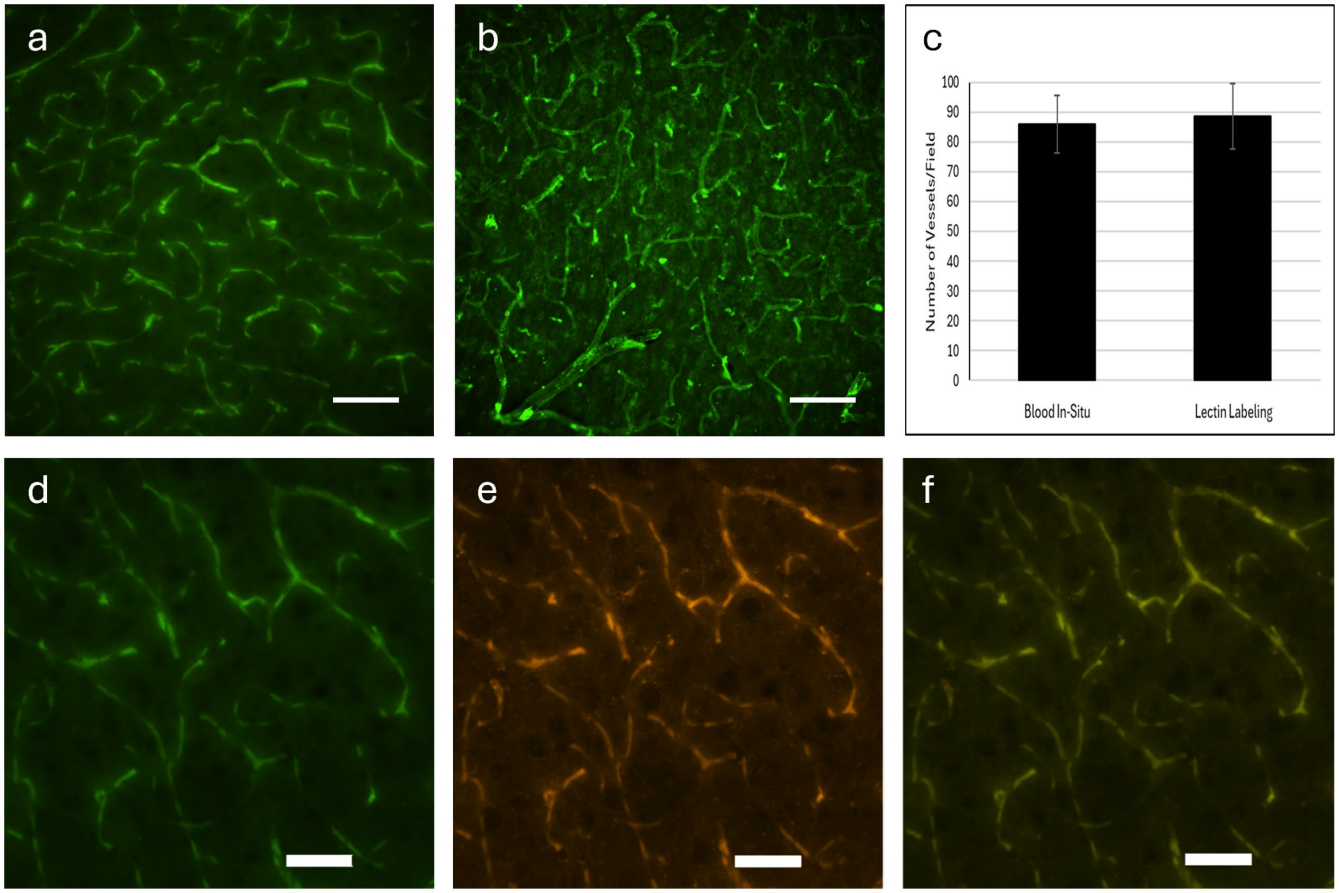
**(a)** Vessels visualized in the prefrontal cortex of a rat using the new method. **(b)** Vessels visualized in the prefrontal cortex of a rat using FITC-conjugated lectin labeling. **(c)** Statistical analysis (Student’s *t*-test) of vessel densities in the prefrontal cortex using these two methods revealed no significant differences between them. **(d)** Image of vessels in the prefrontal cortex of a rat using the new method. **(e)** Image of vessels in the same area as in (**d**) labeled with DyLight594-conjugated lectin. **(f)** Superimposed image of (**d**) and (**e**) showing a perfect match of the double labeling. Scale bars in **(a)** and **(b)** = 100 μm; in (**d**,**e**), and (**f**) = 50 μm.

To determine whether the new method is suitable for vessel density analyses, prefrontal cortex sections from four perfused and four non-perfused rats were used. Five tissue sections from each perfused rat were processed using a conventional lectin histochemistry method, whereas five tissue sections from each non-perfused rat were processed using the new method. Six non-overlapping images from each tissue section were randomly selected and acquired using a 20 × objective for vessel counting. Quantitative analyses of vessel densities determined by the new method (120 images) versus conventional lectin labeling (120 images) revealed no statistically significant difference (Student’s t-test, *p* > 0.05, Figure 4). These results suggest that the new method is adequate for vessel quantification and provides results comparable to those obtained using conventional vessel labeling techniques. Additionally, to provide more definitive validation of the new method—that is, to confirm that blood autofluorescence truly matches the corresponding vessels—we conducted double-labeling experiments using the same tissue sections to image endogenous autofluorescence signals through the FITC filter and DyLight594-conjugated lectin signals through the TRITC filter. In theory, if the new method detects more vessels than those detected by DyLight594-lectin, more “green” vessels should be visible when images of the same area through the respective filters are superimposed. Conversely, if the new method detects fewer vessels than those detected by DyLight594-lectin, more “orange-red” vessels should be visible when images of the same area through the respective filters are superimposed. The experimental results show a complete match of the vessels detected by the double labeling (Figure 4), thereby providing direct proof of the equivalence in vessel detection between the two methods.

## Discussion

The use of blood autofluorescence in biomedical research is not new (Shrirao et al., 2021). In fact, multiphoton microscopy (Kretschmer et al., 2016) and two-photon microscopy (Li et al., 2020) have been used to identify vascular vessels by utilizing blood autofluorescence. However, each of these methods has its own limitations: one has not been validated in brain tissues (Kretschmer et al., 2016), while the other is not suitable for deep brain region imaging (Li et al., 2020). Both methods also require expensive optical imaging systems. This study introduces a completely new approach for detecting cerebrovascular vessels, namely, by visualizing blood autofluorescent signals in submersion-fixed brain samples. The rationale for this approach is that, in submersion-fixed brain samples, blood is fixed *in situ* and remains continuously filling the vessels, as does the blood autofluorescence. To validate the identity of the detected vessels based on autofluorescence signals, we relied on the following experimental results. First, images of the autofluorescence signals resemble the morphological characteristics of cerebrovascular vessels (Figure 1). Second, the autofluorescence signals and the corresponding vessel structures disappeared after washing out the blood through transcardial perfusion (Figure 2a). Third, quantitative vessel density analyses suggest that the new method is as adequate as conventional histochemical labeling using fluorescence-conjugated lectin (Figure 4c). Fourth, the autofluorescence signals detected through the FITC filter completely overlapped with DyLight594-conjugated lectin labeling (Figure 4f). Compared with previous literature utilizing two-photon microscopy and autofluorescence signals to map the arterial and venous networks of the cerebral cortex *in vivo* (Li et al., 2020), which was limited to visualizing vessels only through a cranial window and not in deep brain regions, the new method allows vessel visualization in all brain regions. Compared with conventional histochemical or immunocytochemical vessel labeling techniques, which all require the use of exogenous fluorophores, the new method saves costs by using endogenous autofluorescence signals. Similar to conventional histochemical or immunocytochemical vessel labeling techniques, this new method is also compatible with multiple fluorescent labeling of additional cellular and molecular markers when the appropriate antibodies and/or fluorophores are used. Recent developments in combining tissue clearing and light-sheet fluorescence microscopy techniques enable the labeling, imaging, and analysis of the entire brain vasculature (Zhu et al., 2021; Jiang et al., 2023; Roostalu et al., 2024). Although currently limited to brain tissue sections, this new method requires less time than clearing the entire brain, is more cost-effective than labeling the vasculature using external fluorophores, and does not require a light-sheet microscope, which is an expensive optical system that is not readily accessible to many researchers. In addition, it is suitable for visualizing and analyzing vessels in postmortem human brains, which are valuable resources for biomedical and clinical research. This new method is not meant to replace existing vessel labeling techniques. If brain samples have already been collected following transcardial perfusion, then conventional vessel labeling techniques should be used. However, if a new study has not yet begun, it may be worthwhile to consider this alternative approach to vascular imaging and analysis, based on the abovementioned advantages. Several recent studies combining structural and functional analyses of the cerebrovasculature have demonstrated that mitochondrial ROS and endothelial redox imbalance critically impair neurovascular coupling and cerebral blood flow regulation (Ihuoma et al., 2026) and that vascular oxidative stress contributes directly to cognitive decline and microvascular pathology (Negri et al., 2025; Milan et al., 2026). While our new method is primarily useful for morphological and densitometric analyses of vessels—critical parameters associated with brain development, neurodegeneration, and vascular remodeling—double labeling with cellular and molecular markers, such as GFAP (Figure 2j) and IBA1 (Figure 2k), could help to further focus on specific cell types of the neurovascular unit during these processes. Double labeling with additional molecular markers, such as enzymes and signaling proteins, could help to better understand the molecular mechanisms underlying these processes, thereby facilitating cerebrovascular research in a broader sense.

Although blood vessels could be visualized in postmortem human sections (Figures 2d,e), the number of observed vessels varied among the six subjects. Because of the small sample size, we could not determine whether the variations correlated with different ages, different postmortem processing intervals, disease conditions prior to death, or a combination of these factors. Further research is needed to address this issue. Since blood autofluorescence consists of a complex mixture of autofluorescent molecules in plasma and in different blood cells (Shrirao et al., 2021), it is conceivable that blood degradation could occur during the postmortem processing time and cause the breakdown of certain autofluorescent components, thereby reducing the overall autofluorescent signal intensity in a time-dependent fashion. While a small degradation of blood autofluorescence may not affect vessel analyses using the new method if the blood autofluorescent signals remain strong enough to be detected and distinguished from the surrounding tissue, significant degradation of blood autofluorescence could render vessels undetectable using the new method. Therefore, as with many molecular analyses in postmortem human brains and following established ethical procedures, a shorter postmortem processing interval is preferable. An important finding of this study is that blood vessels can be visualized in postmortem human brain samples without externally applied fluorophores, thus validating the proof-of-concept of this method for human brain samples.

Since blood autofluorescence spans a broad spectrum, it was determined that the predominant autofluorescence signals are through the FITC filter (Figure 2f), while autofluorescence signals through other filters are relatively weak (Figures 2g–i). In our experiments using external fluorophores for co-localization studies, the signal intensities of these externally applied fluorophores were much stronger than those of endogenous autofluorescence through their respective filters. Therefore, under optimal exposure times for these externally applied fluorophores in co-localization studies, there was little interference from endogenous autofluorescence, as shown in Figures 2j,k,l.

The results show that endogenous autofluorescence signals are unevenly distributed in blood vessels in some locations (Figure 3a). This is anticipated, since blood is a highly complex mixture consisting of different types of cells and plasma, which contains a heterogeneous mixture of numerous proteins, hormones, enzymes, and other macromolecules that contribute differently to endogenous autofluorescence signals (Shrirao et al., 2021). However, since blood is fixed *in situ* and continuously fills the vessels, the analyses of vessel density and morphology are not affected by unevenly distributed fluorescent signal intensities due to the continuity of plasma autofluorescence.

Since the new method is based on the autofluorescence of blood, a potential issue may arise under specific conditions, such as blood–brain barrier breakdown or bleeding in the brain—for example, hemorrhagic stroke or traumatic brain injury—since blood extravasation into the parenchymal tissue could interfere with visualization of the vasculature in that area. In fact, virtually every method used for brain vessel analysis is affected by the bleeding issue. Since blood itself is toxic to brain tissue and causes direct injury through physical pressure and the chemical effects of blood breakdown products (Stokum et al., 2021), excessive bleeding is lethal. In such brain samples, it may not be possible to distinguish blood autofluorescence in vessels from that in tissues surrounding the leaked vessels, thereby limiting the use of the new method for vessel analysis in those affected areas. If the bleeding is transient and does not cause death, the leaked blood can eventually be absorbed by glial cells and degraded over time. However, during the recovery phase, before the leaked blood is completely absorbed and degraded, the residue of the leaked blood, with its heterogeneity of autofluorescence signals, could interfere with this new method. Further research on these special cases with blood extravasation is needed to determine the extent of this impact and, ideally, to identify effective solutions.

In conclusion, we anticipate that this method will facilitate cerebrovascular research, particularly research related to vascular morphology and changes associated with disease diagnosis, progression, and treatment outcomes.

## Data Availability

The original contributions presented in the study are included in the article/supplementary material, further inquiries can be directed to the corresponding author.
